# Purtscher-like retinopathy following valsalva maneuver effect: case report

**DOI:** 10.1186/1752-1947-5-338

**Published:** 2011-08-01

**Authors:** Saidin Nor-Masniwati, Yaakub Azhany, Embong Zunaina

**Affiliations:** 1 Department of Ophthalmology, Universiti Sains Malaysia, 16150 Kubang Kerian, Kelantan, Malaysia

## Abstract

**Introduction:**

Purtscher's retinopathy is a rare condition that is noted in cases related to various types of trauma. The characteristic finding in the fundus is the presence of multiple Purtscher flecken. Purtscher-like retinopathy has a similar presentation in the fundus, but without an association with trauma.

**Case presentation:**

A 43-year old Malay man presented with a sudden onset of central foggy vision in the left eye after holding his breath for two minutes while catching a falling object. It was not associated with floaters, flashes of light, or head trauma. His vision in the right eye was 6/6, and in his left eye it was finger counting. He had bilateral temporal sub-conjunctival hemorrhages. An examination of his left fundus revealed multiple white cotton wool spots and dot-blot retinal hemorrhages with diffuse retinal edema at the posterior pole. His right fundus was noted to have only mild temporal peri-papillary edema associated with a few dot-blot hemorrhages. Fundus fluorescein angiography showed good arterial perfusion and no evidence of leaking or neo-vascularization. A diagnosis of Purtscher-like retinopathy was made, and the patient was treated with indomethacin tablets for six weeks. At his six-week follow-up examination, his left eye visual acuity had improved to 6/12. His bilateral sub-conjunctival hemorrhage had resolved. His left fundus showed residual multiple cotton wool spots and reduced retinal edema.

**Conclusions:**

Treatment with non-steroidal anti-inflammatory drugs seems to be effective in reducing edema in patients with Purtscher-like retinopathy.

## Introduction

Purtscher's retinopathy is a rare condition and is noted in cases associated with various types of trauma, including head trauma, seatbelt and airbag pressure, malar bone fracture, crush injury, and chest trauma [1-4]. The fundus characteristics are Purtscher flecken, which are multiple cotton wool spots of varying sizes. The findings in the fundus in patients with Purtscher-like retinopathy are similar, but without any association with trauma. Herein we report the case of a patient with Purtscher-like retinopathy that occurred after the Valsalva maneuver effect.

## Case presentation

A 43-year old Malay man presented to our hospital with sudden-onset blurring of the vision, which he described as central foggy vision that occurred after he caught a piece of falling plywood. He fell to the ground on his buttocks while his left hand was still grasping the plywood and he was holding his breath for about two minutes. Following the incident, he noticed that both his eyes became red and that his left eye vision was reduced. His symptoms were not associated with flashes of light or floaters. He had no complaints of headache, vomiting, abdominal pain, or shortness of breath. He had no direct trauma to the head or loss of consciousness. He is a non- smoker, and he had no significant past medical illness or surgery.

**Figure 1:**
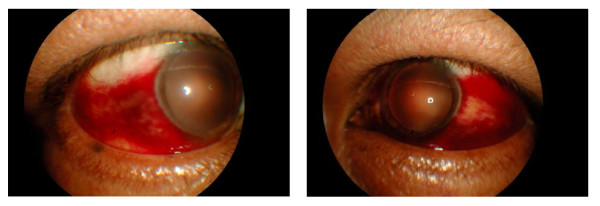
**Photograph showing sub-conjunctival hemorrhage in both eyes at presentation**.

**Figure 2:**
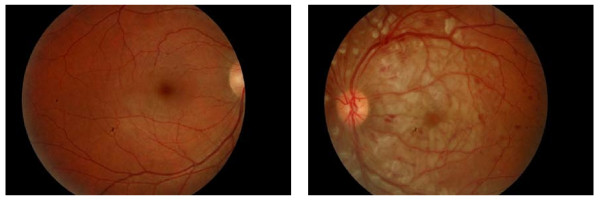
**At the time of presentation, the patient's left fundus (right photograph) had multiple cotton wool spots of various sizes at the posterior pole, and his right fundus (left photograph) had mild temporal peri-papillary edema**.

His right eye vision was 6/6, and his left eye acuity was finger counting, which improved to 6/90 using the pinhole test. He had profound temporal sub-conjunctival hemorrhages bilaterally (Figure 1) but no other significant abnormalities in the anterior segment. The right eye fundus showed mild temporal peri-papillary edema associated with a few dot-blot hemorrhages. An examination of his left fundus revealed peri-papillary and macular edema with multiple dot-blot hemorrhages and cotton wool spots at the posterior pole (Figure 2). His systemic examination revealed no abnormality.

Fundus fluorescein angiography (FFA) showed good arterial perfusion with no leaking area or neo-vascularization. Blood investigations for complete blood count, serum amylase, and clotting time were normal. His chest X-ray revealed no significant findings.

**Figure 3:**
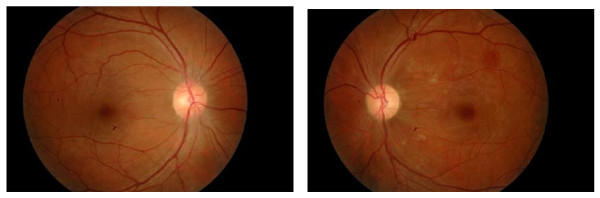
**The patient's left fundus (right photo) showed resolving cotton wool spots, and his right fundus showed resolved temporal peri-papillary edema at the six-week follow-up examination**.

A diagnosis of Purtscher-like retinopathy following a Valsalva maneuver was made. He was treated with indomethacin tablets 25 mg daily for six weeks. At his six-week follow-up examination, the visual acuity of his left eye had improved to 6/12. His sub-conjunctival hemorrhages in both eyes and his right eye temporal peri-papillary edema had resolved. The left fundus appeared to have residual macular edema and resolving cotton wool spots (Figure 3).

## Discussion

Purtscher's retinopathy is a rare condition that was first described in 1910 by Otmar Purtscher. Purtscher encountered a case of traumatic retinopathy secondary to head trauma which was associated with sudden blurring of vision [1]. Later a similar finding was noted in cases related to various types of trauma, including seatbelt and airbag pressure, malar bone fracture, crush injury, and chest trauma [1-4]. Purtscher-like retinopathy is seen in patients with acute pancreatitis, systemic lupus erythematous, HELLP syndrome, and renal failure, as well as in patients with adenocarcinoma of the pancreas with no relation to trauma [5-8].

The diagnosis of Purtscher's retinopathy is made according to the patient's history and clinical presentation. Patients usually experience asymmetrical symptoms in both eyes, and the symptoms can also be unilateral. The characteristic findings in the fundus are Purtscher flecken, which are multiple cotton wool spots of varying sizes. FFA is performed to look for arterial occlusion and areas of capillary leakage [9].

The pathogenesis of Purtscher's retinopathy is still uncertain. Lin *et al. *[2] proposed that an increase in thoracic pressure leads to a reflux in the venous system, which causes endothelial damage. This sequence of events leads to incompetence of the microvascular circulation, resulting in occlusion and ischemia. Agrawal and McKibbin [9] suggested a few potential mechanisms related to this condition, such as increased intra-cranial pressure and lymph extravasation, increased intra-thoracic pressure and venous dilatation, vasculitis due to free fatty acids, and vascular emboli caused by air, fat, leukocytes, fibrin, platelets, and complement activation. In our patient, the effect of the Valsalva maneuver might have been the cause of his Purtscher-like retinopathy.

There are no specific recommended guidelines for the treatment of patients with Purtscher's retinopathy [4]. Wang *et al. *[10] reported a case of a patient who was given a mega-dose of steroid that showed a good visual response within the first two weeks of treatment. A patient who had sustained a chest contusion who was treated with 39 courses of hyperbaric oxygen twice a week led to gradual improvement of visual function and retinal appearance [2]. We treated our patient with oral indomethacin 25 mg/day for six weeks. A previous report described indomethacin therapy that led to improvement of visual acuity in a patient with chronic cystoid macular edema after a cataract operation [11]. Non-steroidal anti-inflammatory drugs (NSAIDs) act as cyclooxygenase inhibitors and thus reduce the formation of endogenous prostaglandins.

Holak and Holak [12], on the basis of optical coherence tomography examinations, suggested that acute retinal changes of more than eight weeks' duration in patients with Purtscher's and Purtscher-like retinopathy may carry a poor prognosis. A retrospective study done by Agrawal and McKibbin [9] in response to the study by Holak and Holak [12] suggested that there is a relation between the delayed resolution of acute vision changes and poor outcomes, but their findings were not statistically significant. Our patient presented soon after the precipitating incident and was started on treatment immediately. His visual outcome had improved to 6/12 by the time of his six-week follow-up examination.

## Conclusions

In our patient, NSAID therapy was effective in treating Purtscher-like retinopathy.

## Consent

Written informed consent was obtained from the patient for publication of this case report and any accompanying images. A copy of the written consent is available for review by the Editor-in-Chief of this journal.

## Competing interests

The authors declare that they have no competing interests.

## Authors' contributions

SNM, YA, and EZ examined and evaluated the patient. SNM wrote the manuscript. EZ edited the manuscript. All authors read and approved the final manuscript.
